# Differential Expression and Prognostic Correlation of Immune Related Factors Between Right and Left Side Colorectal Cancer

**DOI:** 10.3389/fonc.2022.845765

**Published:** 2022-07-22

**Authors:** Yue Hu, Jie Ding, Chengjiang Wu, Hong Gao, Meiling Ge, Qixiang Shao, Yanhong Liu, Qing Ye

**Affiliations:** ^1^ Biobank of Nanjing Drum Tower Hospital, The Affiliated Hospital of Nanjing University Medical School, Nanjing, China; ^2^ Department of Clinical Laboratory, The Second Affiliated Hospital of Soochow University, Suzhou, China; ^3^ Jiangsu Key Laboratory of Medical Science and Laboratory Medicine, Department of Immunology, School of Medicine, Jiangsu University, Zhenjiang, China; ^4^ Department of Pathology, The First Affiliated Hospital of University of Science and Technology of China (USTC), Division of Life Sciences and Medicine, University of Science and Technology of China, Hefei, China; ^5^ Intelligent Pathology Institute, Division of Life Sciences and Medicine, University of Science and Technology of China, Hefei, China

**Keywords:** colorectal cancer, immune-related factors, left-side colorectal cancer, right-side colon cancer, differential expression, prognostic correlation

## Abstract

**Background:**

Growing evidence suggests that colorectal cancer (CRC) should be considered a heterogeneous disease. The right side (RCC) and left side (LCC) colorectal cancer have different clinical characteristics and immune landscapes. The aim of this study was to analyze differential expression and prognostic correlation of immune-related factors between RCC and LCC.

**Methods:**

The gene expression profile and clinical characteristics of CRC patients were retrieved from The Cancer Genome Atlas data portal (n=525). Using a deconvolution algorithm, immune cell infiltration in RCC and LCC based on the RNA-seq data was analyzed. Differentially expressed genes (DEGs) were obtained by performing differential gene expression analysis. Immune-related DEGs were derived by the intersection with immune-related factors downloaded from the IMMPORT database. To further validate the findings, we applied immunohistochemical (IHC) staining of a CRC tissue microarray (TMA). The distribution of immune cells in RCC and LCC and changes in the expression of immune molecules on their membranes were verified. The expression levels of circulating cytokines were measured by flow cytometry to detect the cytokines secreted by immune cells in RCC and LCC. Furthermore, to reveal the prognostic value of differential immune factors on RCC and LCC patients, survival analysis based on mRNA levels using TCGA cohort and survival analysis using protein levels was performed using our CRC patients.

**Results:**

The infiltration of immune cells differed between RCC and LCC, the infiltration degree of macrophages M0 was significantly higher in LCC, while the infiltration degree of differentiated macrophages M1 and M2, CD4+ T and CD8+ T cells was significantly higher in RCC. The expression of related molecules by immune cells also differed between RCC and LCC. The expression of 7 genes in RCC was higher than that in LCC, which were CCR5, CD209, CD8A, HCK, HLA-DPB1, HLA-DQA1, HLA-DRA, respectively. Meanwhile, the expression of 2 genes in LCC was higher than in RCC, which were IL-34 and PROCR. Patients with RCC having high expression of HLA-DQA1 mRNA or proteins had better survival and LCC patients with high expression of IL 34 mRNA or protein had better survival.

**Conclusions:**

In this study, we comprehensively compared differences in immune cells and regulating factors between left and right colorectal cancer. Different expression patterns and their effects on survival were identified. The analysis of immune-related factors may provide a theoretical basis for precise immunotherapy of RCC and LCC.

## Introduction

Colorectal cancer (CRC) is one of the most common malignant tumors. Its incidence rate ranks third in the world and the mortality rate is ranked second. CRC is a malignant tumor of the digestive system, and is the first tumor in the world in terms of morbidity and mortality and seriously threatens life and health of individuals (1). The colon is a remarkable organ as it manages the final products of digestion after nutrient assimilation and packages it as waste to expel. Fundamentally, the colon is one organ but develops from two different embryonic areas of the primitive gut: the midgut, which gives rise to the small intestine through the proximal two-thirds of the transverse colon, and the hindgut, which gives rise to the distal third of the transverse colon through the upper anal canal (2). The colon is divided into left-sided and right-sided colons by the splenic flexure. Right-sided tumors are classified as originating proximal to the splenic flexure (cecum, ascending colon, transverse colon), while left-sided tumors arise distally to this site (descending colon, sigmoid colon, and rectum) (3). Statistics show that the incidence of left and right-sided colon cancer differs according to sex and age groups. Patients with right-sided cancer are more likely to be older, women, diagnosed with a more advanced stage, and have more poorly differentiated tumors. Studies have reported that the proportion of women with right-sided colon cancer (RCC) and left-sided colon cancer (LCC) is 62% and 52%, respectively. In addition, the proportion of older patients (age≥75 years) with RCC (69%) is significantly higher than in LCC (61%) (4).

Despite advances in screening, diagnosis, and curative resection, CRC remains one of the leading causes of cancer‐related deaths worldwide, and its clinical outcome for individual cases remains unsatisfactory (1, 5). As a well‐recognized heterogeneous disease, the phenotype and prognostic diversity of CRC present great challenges in making individualized clinical decision, especially for right- and left-side CRC. A growing number of studies have shown that there are differences in molecular biological characteristics between RCC and LCC (6, 7). For example, the probability of abnormal chromosome copy number and structural rearrangement of LCC is significantly higher than that of RCC (8). The probability of the CpG island methylator phenotype (CIMP) in RCC is significantly higher than in LCC (9). However, malignant cancer phenotypes are defined not only by intrinsic activities of tumor cells, but also by immune cells recruited and activated in the tumor-related microenvironment (10). Studies have shown that there are differences in tumor infiltrating immune cells (TIICs) between LCC and RCC, with higher expression of CD8+ T cells in RCC and higher expression of CD56^bright^ NK cells in LCC (11). However, immune cells play a role through immune-related factors, and these immune-related factors are easier targets for diagnosis and treatment than immune cells, and play an important role in the diagnosis, treatment, prediction of prognosis, and follow-up monitoring of CRC. To date, few studies have investigated differences in immune-related factors and the immune microenvironment in LCC and RCC. In this study, we comprehensively compared differences in the expression of immune related genes between right- and left-side CRCs. In addition, we explored the differences in T cells infiltration, systemic cytokines levels and the impact of gene expression on survival.

## Materials and Methods

### Ethics Statement

The studies involving human participants were reviewed and approved by the Ethics Committee of Nanjing Drum Tower Hospital. The participants provided their written informed consent to participate in this study.

### Data Sources

A total of 2498 immune-related genes were downloaded from the IMMPORT database (https://www.immport.org/home). Gene expression profiles were downloaded from The Cancer Gene Atlas (TCGA) database and included a total of 525 tissue samples including 207 LCC and 318 RCC tissue samples (updated December 31, 2017; https://portal.gdc.cancer.gov/). The clinical data downloaded from CRC included survival time, survival status, age, sex, stage, classification, tumor site, and other information.

### Analysis of Gene Differential Expression in mRNA

Expression profiles downloaded from TCGA were analyzed using R. The expression profiles were standardized and the limma package was used to identify differentially expressed genes (DEGs), which were crossed with immune-related genes downloaded from the IMMPORT database to obtain immune-related DEGs and to construct a heatmap.

### Survival Analysis From the TCGA Database in mRNA

To reveal the prognostic value of differentially expressed genes in patients with RCC and LCC, survival analysis was performed. The gene expression values and clinical data of CRC were downloaded from the TCGA database. A total of 525 samples were included. The samples in the data were divided into RCC and LCC groups, the high expression and low expression group according to the median value of each differential gene. We performed a survival analysis for all nine DEGs, including HLA-DQA1, CCR5, CD209, CD8A, HCK, HLA-DPB1, HLA-DRA, IL-34, and PROCR, to observe the correlation with patient survival.

### Analysis of Immune Cell Infiltration From the TCGA Database in mRNA

The expression profiles and clinical data of CRC were downloaded from TCGA database, and immune cell infiltration was analyzed. The CIBERSORTx web tool was used to characterize the abundance of 22 immune cell types based on the RNA-seq data in RCC and LCC. Using a deconvolution algorithm (12). The CIBERSORTx analysis identified the profiles of 22 immune cell types including B cells, T cells, natural killer (NK) cells, macrophages, and dendritic cells (DC). Differences in immune cell infiltration of CD4+ T cells, CD8+ T cells, macrophages M0, M1, and M2 between RCC and LCC were investigated and analyzed.

### Biospecimens and Clinicopathological Analysis

For the analysis of clinical tissues, 200 paraffin-embedded CRC tissues samples derived from CRC and normal mucosal tissues from 100 patients with colon cancer were prepared. Of these 100 patients, there were 50 patients with RCC and 50 patients with LCC. Furthermore, the pathological diagnosis of all samples was confirmed by two pathologists, and relevant clinical and pathological information was collected.

To measure circulating cytokines levels in patients with either RCC or LCC, 0.5 mL preoperative plasma samples from 29 patients with CRC (17 LCC and 12 RCC) were collected. Biospecimens were collected from the biobank of Nanjing Drum Tower Hospital, the Affiliated Hospital of Nanjing University Medical School during 2013- (2019)

### Immunohistochemical for Validation of Gene Expression Data

We collected 200 pairs of paraffin tissue samples, consisting of tumor tissue and normal colonic mucosa. These 200 paraffin tissues were used to prepare a tissue microarray with 50 targets in each TMA, and a total of 4 TMAs were made. For each case, a 1.5-mm diameter core was sampled from the tumor areas. An additional 1.5-mm diameter core was taken from the histologically normal colonic mucosa samples. Sequential 3 μm-sections were prepared by experienced pathology technicians.

Whole TMAs were stained using the standard protocol for HE and immunohistochemistry. Briefly, tissues were deparaffinized, hydrated, and endogenous peroxidase activity was blocked with a solution of 3% methanol in hydrogen peroxide for 10 min. Following antigen retrieval in a water bath at 98°C with Tris EDTA, pH 9 or citrate buffer, pH 6. The TMAs were incubated overnight at 4°C with specific antibodies, then incubated at room temperature with secondary antibodies (TYPING, TPB0050) for 20 min and 3-3’-diaminobenzidine for 3-5 min, then were counterstained with hematoxylin for 30 min. The results were determined and scored according to the proportion and intensity of the staining of positive cells. TMAs test with specific antibodies against HLA-DQA1 (abcam, ab128959), IL-34 (abcam, ab224734), CD83 (abcam, ab2015343) as a marker for mature DC cells, TCRα (abcam, ab18861), TCRβ (Novus, NBP2-22486), and TCRγδ (BioLegend, 331202).

The heterogeneity of the tumor and the expression of some immune-related molecules varies between the adjacent and normal regions of the tumor. Whole paraffin sections of tumor, paratumor and normal region corresponding to TMA patients (50 LCC and 50 RCC) were used for immunohistochemical detection of CD80 (abcam, ab134120) as a marker for M1 cells, and CD163 (abcam, ab182422) as a marker for M2 cells.

### Detection of Circulating Cytokines

Plasma samples from CRC patients were prepared for flow cytometry. The cytokines IL-2, TNF-α and IFN-γ secreted by Th1 cells, IL-4, IL-6, and IL-10 secreted by Th2 cells, and IL-17A secreted by Th17 cells were detected using the Cytometric Bead Array (CBA) TH1/TH2/TH17 cytokine kit (CBA Human Th1/Th2 cytokine kit, CEGER, China, http://cell-genebio.com) and the FACSCanto II flow cytometer (BD, USA). Specific steps were as follows: venous blood samples were collected using an EDTA anticoagulant tube, centrifuged at 2000-4000 rpm for 20 min, and the plasma supernatant was retained for later use. The mixed solution of the captured microspheres was centrifuged at 200 ×g for 5 min in a low speed centrifuge and the microsphere buffer solution of the same volume as the supernatant was added. After fully mixing, the samples were incubated in the dark for 30 minutes. After incubation, the solution was mix well, and 25 μL was added to each experimental tube. A 25 μL-volume of standard solution was added to the sample tube followed by 25 μL of fluorescence detection reagent. After fully mixing, the samples were incubated at room temperature in the dark for 2.5 h. Next, 1 mL PBS solution was added to each tube and centrifuged at 200 ×g for 5 min. The supernatant was carefully aspirated away and 100 μL pf PBS solution was added to each tube and allowed stand for before testing. Based on the data obtained from the test, the standard curve was drawn, and the content of each cytokine in the sample was extracted based on the constructed standard curve.

### Survival Analysis of CRC Patients

Clinical follow up of 100 patients with CRC, the survival status and survival time of the patients were surveyed by telephone follow-up. The follow-up period was 1 year and the median follow-up time was 2178 days. Survival analysis was performed in combination with the results of IHC protein expression.

### Statistical Analysis

Continuous variables were analyzed using t-tests or nonparametric rank sum tests. Categorical variables were analyzed using Chi-square tests. Prognostic analyses were performed using Kaplan-Meier survival analysis. All data were analyzed using GraphPad Prism 8 for Windows (GraphPad Software Inc., San Diego, CA, USA) and R 3.6.3 (https://www.r-project.org/). Results with P<0.05 were considered statistically significant.

The P-values were obtained from univariate Cox proportional hazards regression models using GraphPad Prism 8. All Kaplan-Meier survival curves were drawn using the GraphPad Prism 8 and plotted together with the x-axis representing time, and ranging from 0 to 5000 days. P<0.05 was considered statistically significant.

## Results

### Clinicopathological Characteristics of the Study Population

We analyzed the clinical and pathological data of 525 samples downloaded from the TCGA database. We found that RCC and LCC patients differed in age, pathology stage, mucus secretion, and microsatellite stability. The baseline characteristics according to tumor location are summarized in **Supplementary Table 1**. Compared with LCC, RCC has clinicopathological features of advanced age (≥70 years), less lymph node metastasis, mucus secretion, and microsatellite instability. These findings were consistent with those from previous studies (13, 14), which shows that there is no bias in our study population.

Analysis of the clinical and pathological information of the 100 patients enrolled revealed that RCC and LCC patients differed in terms of age, pathologic stage, and microsatellite stability, which was consistent with TCGA data (**Supplementary Table 2**). Of note, due to population differences, pathology M stage differed between the two sets of data, but there was no significant difference in the final pathology stage.

### Differential Analysis of Immunologically-Relevant Gene Expression in RCC and LCC

**Figure 1:**
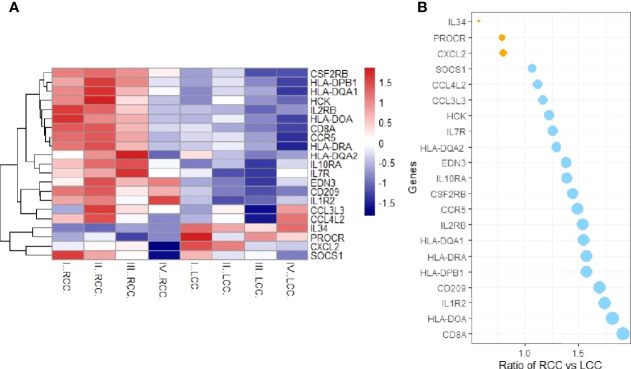
Immunologically relevant differential genes expression analysis. **(A)** Heatmap of immune-related differential genes in RCC and LCC. The X-axis represents LCC and RCC simples at different stages, and the Y-axis represents immune-related genes. Red means high expression and blue means low expression. CCR5, CD209, CD8A, HCK, HLA-DPB1, HLA-DQA1 and HLA-DRA were highly expressed in RCC, while IL-34 and PROCR were highly expressed in LCC. **(B)** Bubble chart of the expression ratios of the DEGS in RCC and LCC.

The expression profile data downloaded from TCGA were processed. The probe names were converted to gene names, the data were standardized and then the differential expression analysis was performed using the R package ‘limma’. We then compared the differentially expressed genes obtained with immune-related genes downloaded from IMMPORT, and screened for immune-related DEGs, and a heat map was drawn (**Figure 1A**). The overall expression of immune-related genes in RCC was higher, while in the LCC, lower expression was observed especially in stage I, II, III, and IV samples. Compared to the LCC, the genes with higher expression in RCC included: CCR5, CD209, CD8A, HCK, HLA-DPB1, HLA-DQA1 and HLA-DRA. Genes with higher expression in LCC were IL-34 and PROCR, compared to the RCC. There was no significantly expression difference of CXCL2 especially in each stage samples compared with IL34 and PROCR according to the heatmap and bubble chart. These genes were selected for further study. The ratios of gene expression in RCC *vs* LCC greater than 1 are shown in blue, and those smaller than 1 in orange (**Figure 1B**).

### Differentially Expressed Genes in mRNA Are Related to Patient Survival

**Figure 2:**
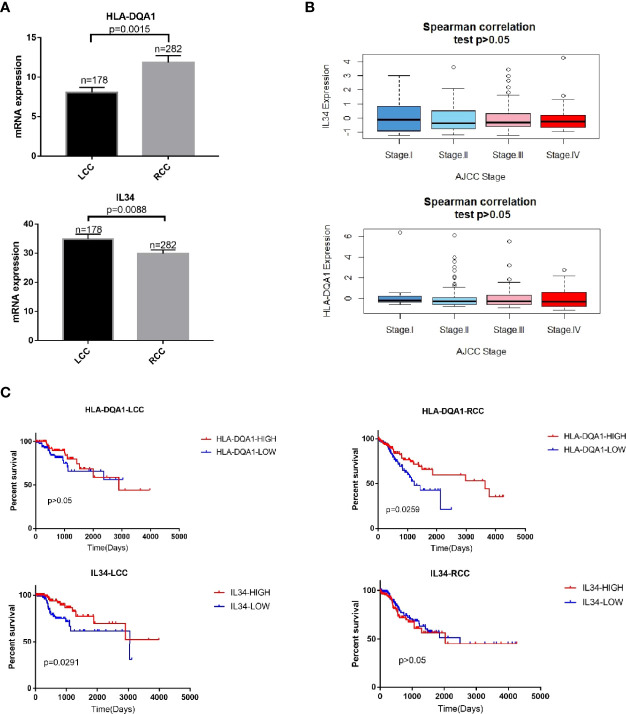
mRNA expression of immune-related DEGs are related to patient survival. **(A)** HLA-DQA1 had higher expression in RCC and IL-34 showed higher expression in LCC. **(B)** Spearman correlation analysis. There was no significant correlation between IL-34/HLA-DQA1 and different stages, P>0.05. **(C)** Survival analysis showed that patients with high HLA-DQA1 expression had significantly prolonged survival in RCC, P=0.0259. HLA-DQA1 expression had no significant effect on survival in LCC, P>0.05. Patients with high IL-34 expression significantly prolonged their survival time in LCC, P=0.0291. IL-34 expression had no significant effect on survival in RCC, P>0.05.

The expression of HLA-DQA1 was higher in RCC. The RCC and LCC samples were divided into high and low expression groups according to the median expression of HLA-DQA1 and IL-34, respectively. To further study the relationship between DEGs and tumor stages, Spearman correlation analysis was performed. There was no significant correlation between DEGs and different tumor stages (**Figures 2A, B**).

In RCC where expression of HLA-DQA1 was higher than LCC, survival analysis showed that patients with RCC with higher expression of this molecule had prolonged survival times. Meanwhile, in LCC where expression of IL-34 was higher than RCC, survival analysis showed that patients with LCC with higher expression of this molecule had prolonged survival times (**Figure 2C**). Similarly, survival analysis was performed for the other 8 genes with left and right differences and CXCL2, the results were not statistically significant (**Supplementary Figure 1**). We selected HLA-DQA1 and IL-34 for further study.

### Protein Expression and Prognostic Correlation of HLA-DQA1 and IL-34

**Figure 3:**
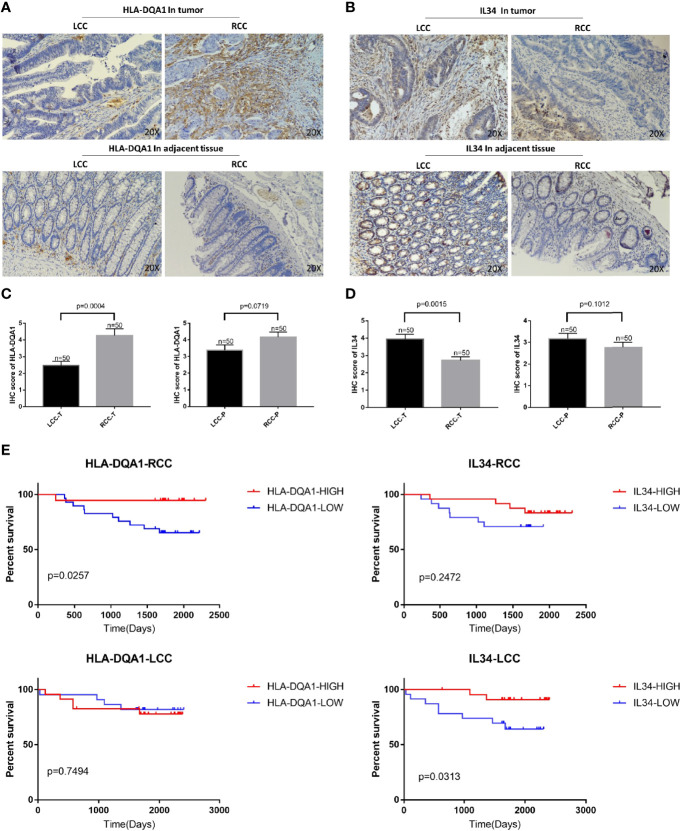
Differential expression of HLA-DQA1 and IL-34 proteins in LCC and RCC are associated with survival. The IHC images were taken at ×200 magnification. **(A, C)** The expression of HLA-DQA1 in tumor tissues of the RCC was significantly higher than that of the LCC, there was no difference in the paracancer tissue between LCC and RCC. **(B, D)** The expression of IL-34 in tumor tissues not the adjacent tissue of the LCC was significantly higher than that of the RCC. **(E)** Survival analysis of follow-up results are consistent with the results in **Figure 2**.

Antibodies of HLA-DQA1 and IL-34 were used for IHC staining. As shown in **Figures 3A–D**, HLA-DQA1 expression in the tumor tissues of the RCC was significantly higher than that of the LCC. However, the expression of IL-34 in LCC tumor tissues was significantly higher than in RCC. The above results confirmed the difference in HLA-DQA1 and IL-34 expression in LCC and RCC through validation of clinical samples and verified the results of the data analysis.

Survival analysis of the follow-up results of the 100 patients showed that increased expression of HLA-DQA1 significantly prolonged survival in RCC, while increased expression of IL-34 significantly prolonged survival in LCC, consistent with the results of previous data analysis (**Figure 3E**).

### Analysis of Differences in Immune Infiltration in RCC and LCC

**Figure 4:**
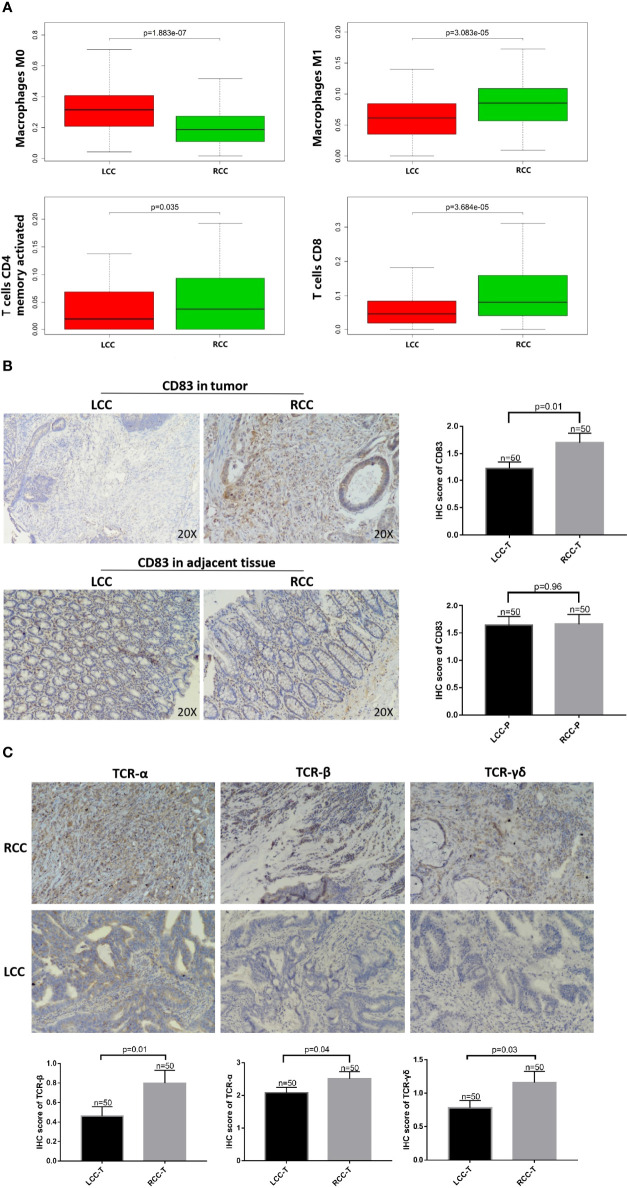
High HLA-DQA1 expression in RCC promotes antigen presentation and cytotoxic activity. The IHC images were taken at ×200 magnification. **(A)** Differential analysis of TIICs showed that CD4+ T, CD8+ T and macrophages M1 were more expressed in RCC, while macrophages M0 was more expressed in LCC. **(B)** CD83, a marker of mature DCs, was used for IHC staining and it was highly expressed in tumor tissues of RCC but not LCC. **(C)** TCRαβ and TCRγδ were both more expressed in RCC than in LCC.

Using CIBERSORTx, the immune infiltration of 22 types of cells, including B cells naive, B cells memory, plasma cells, T cells CD8, T cells CD4 naive, T cells CD4 memory resting, T cells CD4 memory activated, T cells follicular helper, T cells regulatory (Tregs), T cells gamma delta (TCRγδ), resting NK cells, activated NK cells activated, monocytes, M0 macrophages, M1 macrophages, M2 macrophages, resting dendritic cells, activated dendritic cells, resting mast cells, activated mast cells, eosinophils, and neutrophils was deconvoluted. The results showed that there was a significantly higher degree of infiltration of M1 macrophages, CD4+ memory cells, CD8+ T cells, resting mast cells, and T cells follicular helper in RCC compared to LCC. Meanwhile, there was a significantly higher degree of M0 macrophages, activated mast cells, infiltration in LCC compared to RCC. There was no significant difference between RCC and LCC in the degree of infiltration of other types of immune cells (**Figure 4A**; **Supplementary Figure 2**). It should be noted that the expression of B cells memory, T cells gamma delta, monocytes, activated dendritic cells, eosinophils and T cells CD4 naive were very low and there was no statistical difference, so these figures were not shown.

### Antigen Presentation Related Molecules CD 83, TCRαβ, and TCRγδ Were Elevated in RCC

IHC staining was performed using CD83 antibody, a marker of mature DCs (15, 16). CD83 expression was significantly higher in RCC than in LCC (**Figure 4B**). Differences in immune cell infiltration between RCC and LCC using RNAseq data of TCGA cohort showed that CD4+ memory cells and CD8+ T cells were significantly more expressed in RCC than in LCC (**Figure 4A**). To further study the role of antigen presentation, IHC experiments using TMA showed that TCRαβ and TCRγδ were both more expressed in the RCC than in the LCC, the difference was statistically significant. (**Figure 4C**).

### The Expression of IL-6, and IL-10 Was Elevated in LCC

**Figure 5:**
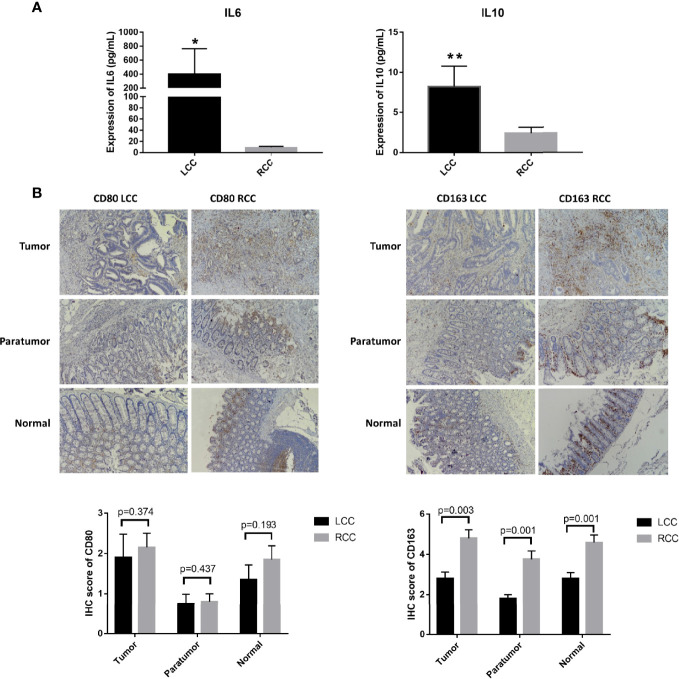
Differences in expression of cytokines and macrophage surface markers in LCC and RCC. **(A)** Flow cytometry analysis of cytokines showed that TH2 cytokines IL-6 and IL-10 were highly expressed in LCC. **(B)** The IHC images were taken at ×200 magnification. The infiltration of CD163+ cells was higher in tumor, paratumor and normal tissues of RCC (n=20, p<0.05). The symbol * show p<0.05, the specific value is "p=0.0207". The symbol ** show p<0.01, the specific value is "p=0.0059".

Plasma samples were used for flow cytometry. The cytokines IL-2, IL-4, IL-6, IL-10, TNF, IFN and IL-17A were measured using the CBA cytokine kit. Cytokine analysis showed that IL-6 and IL-10 were significantly elevated in patients with LCC compared to those with RCC. Low expression of IL-4 and IL-17A was detected in patients with LCC with concentrations of 0.3pg/mL and 0.9pg/mL, respectively. IL-4 and IL17A were not detected in patients with RCC. IL-2, TNF and IFN were not detected in patients with LCC and RCC. So there was no statistical difference in the 5 cytokines (**Figure 5A**, **Table 1**).

**Table 1:** The expression of cytokines in LCC and RCC. Flow cytometry analysis of TH1, TH2 and TH17 subtype cytokines showed that IL-6 and IL-10 were highly expressed in LCC, P<0.05.

Type	n	IL2(pg/mL)	IL4(pg/mL)	IL6(pg/mL)	IL10(pg/mL)	TNF(pg/mL)	IFN(pg/mL)	IL17A(pg/mL)
**LCC**	17	0	0.3	398.1(0.6-6224.5)*	8.2(0.6-44.3)**	0	0	0.9
**RCC**	12	0	0	8.6(0.6-30.6)	2.4(0.2-8.8)	0	0	0

Mean (Minimum-Maximum), *show p<0.05, **show p<0.01.

### Differences in Macrophage Infiltration and Expression of Their Surface Markers in LCC and RCC

Differential analysis of immune cell infiltration in RCC and LCC using RNAseq data from TCGA cohort revealed that significantly higher number of macrophages M0 were infiltrated in LCC, while higher number of differentiated macrophages M1 were infiltrated in RCC (**Figure 4C**). The biomarkers for M1 and M2 were CD80 and CD163 (17–19). IHC staining showed infiltration of CD163+ cells was higher in RCC and infiltration of CD163+ cells was higher in tumor, paratumor and normal tissues of RCC. The infiltration of CD80+ cells was not significantly different in tumor, paratumor, and normal tissues of patients with RCC and LCC. (**Figure 5B**; **Table 2**).

**Table 2:** Differences in macrophage infiltration and expression of their surface markers in LCC and RCC. THE high infiltration of CD163 was significantly different in tumor, paratumor and normal tissues of RCC, P<0.05.

Region	Marker	LCC, n=20	RCC, n=20	p value
**Tumor**	**CD80**	1.9	2.15	0.374
	**CD163**	2.8	4.8	0.003
**Paratumor**	**CD80**	0.75	0.8	0.437
	**CD163**	1.8	4.75	8.813E-05
**Normal**	**CD80**	1.35	1.85	0.193
	**CD163**	2.8	3.6	0.001

## Discussion

In recent years, tumor immunotherapy has become an important focus of research. Therefore, understanding the tumor immune environment and the expression of immune-related factors is helpful for improving immunotherapy approaches. Immune-related factors play a significant role in tumor progression and immunotherapy (20), which warrants further study of the immune mechanisms involved. Different immune mechanisms of RCC and LCC have been reported, but systematic studies on differences between immune related factors of RCC and LCC have are currently unknown. Previous studies have examined the differences between RCC tumor-infiltrating immune cells and LCC (11, 21), but additional studies on immune-related factors will help identify molecular targets and provide a basis for clinical immunotherapy.

The formation of the tumor immune microenvironment is the result of the interaction between tumor cells, immune cells, and nonimmune stromal cells (including fibroblasts and endothelial cells), which play a key role in the occurrence and progression of tumors. The immune system is particularly composed of innate immune cells such as neutrophils, macrophages, dendritic cells, mast cells and natural killer cells, and adaptive immune cells such as T and B lymphocytes, participating in prevention and promotion of tumor development, having pro and anti-tumor functions (22–26). Therefore, relevant molecular markers of antitumor immunity and the regulatory pathway status of antitumor activity is reflected in the tumor immune microenvironment under pathological conditions and can be used as indicators to predict tumor prognosis, Furthermore, factors that play a key role in the mechanism of tumor immune escape can be used as targets for precision tumor therapy.

In this study, the differential expression of immune-related factors in LCC and RCC was investigated. Data on clinical and gene expression profiles related to CRC were downloaded from TCGA database. First, clinical information was analyzed to identify differences between the clinicopathological characteristics of RCC and LCC. Statistics showed that the proportion of female and elderly patients with RCC was higher, histology was often manifested as low differentiation, adenocarcinoma with mucus secretion, and MSI was more common. Analysis of gene expression profile data identified a total of seven immune-related genes that are highly expressed in RCC: CCR5, CD209, CD8A, HCK, HLA-DPB1, HLA-DQA1, and HLA-DRA. However, the immune-related genes that were highly expressed in LCC were IL-34 and PROCR. Further survival analysis revealed that HLA-DQA1 was highly expressed in RCC and was associated with a good prognosis. IL-34 was highly expressed in LCC and was associated with a prolonged survival of patients. Several other genes were not significantly associated with patient survival. IHC staining methods were used to further verify the expression of these two genes in clinical samples, confirming the results of data analysis. Survival analysis of follow-up results from the 100 clinical cases showed that increased expression of HLA-DQA1 significantly prolonged survival in RCC, while increased expression of IL-34 significantly prolonged survival in LCC. To further analyze the regulatory role of IL-34 and HLA-DQA1 in LCC and RCC, we analyzed differences in immune cell infiltration, antigen presentation, and cytokine secretion in LCC and RCC.

**Figure 6:**
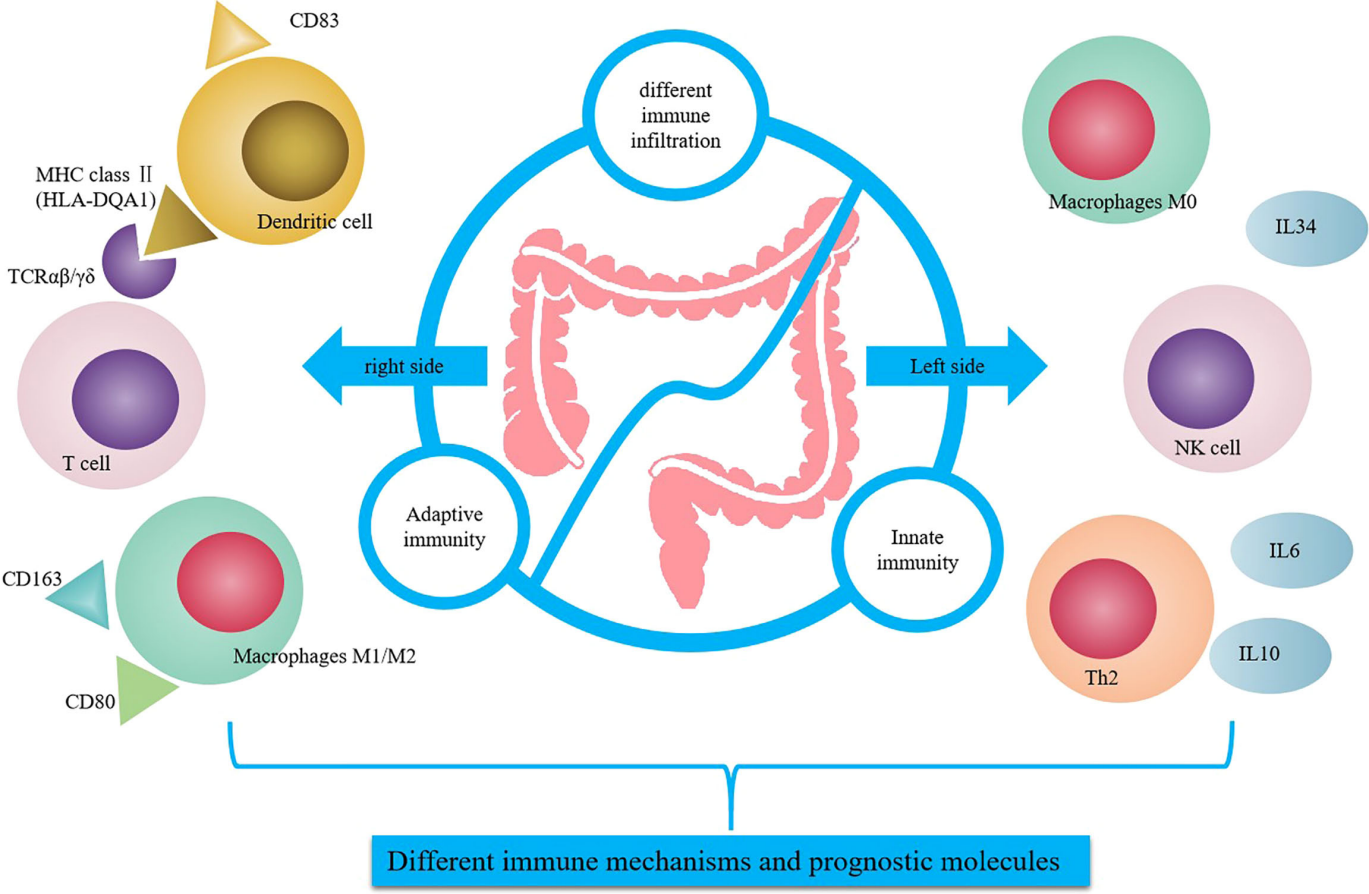
Summary of differential regulation of immune related genes between RCC and LCC. HLA-DQA1 was secreted by mature DC and combined with TCR to enhance the antigen presentation and cytotoxic activity in RCC. IL-34 was secreted by macrophage M0, endothelial cells or other cells to promote cytokines secretion and NK cells activity in LCC.

The results showed that HLA-DQA1 was highly expressed in RCC, and CD4+T, CD8+T, M1 and M2 cells were highly infiltrated immune cell populations, and TCR expression was high, and antigen presentation was strong. HLA-DQA1 is a member of the MHC class II family, located on chromosome 6p21, and may be a potential prognostic biomarker for esophageal squamous cell carcinoma (27). One of the primary functions of MHC class II molecules in the immune system is to present antigens from extracellular proteins to CD4+ T cells for recognition. HLA-DQA1 is expressed by specialized antigen presenting cells (APCs), including DCs, mononuclear macrophages, and B cells. Abnormal expression of MHC II can lead to an inadequate immune response or autoimmune responses, leading to a variety of diseases, including cancer (22, 28). IHC staining showed that antigen presentation related molecules CD83, TCRαβ and TCRγδ were elevated in RCC. The CD83 molecule is expressed by a great variety of cells types, these include monocytes, macrophages, B cells, activated CD4+T cells and so on (29).. The CD83 expression on some of the tissue sections is not entirely due to the infiltration of DCs, which may be other cells. The natural antigen is modified intracellularly through metabolism and binds to MHC class II to form the antigenic peptide-MHC II molecule complex. The complex is recognized by the TCR of antigen-specific T cells. TCRs can be divided into TCRαβ and TCRγδ according to the composition of dipeptide chains, among which 95% is TCRαβ. The CIBERSORT analysis showed that the expression of TCRγδ was very low, and there was no statistical difference although the expression is higher in RCC. The CIBERSORT analysis is based on the bulk transcriptome and IHC analysis is based on protein expression. So we found differences between RCC with LCC at both the RNA and protein levels but the difference at protein level was even more pronounced. Mature DCs, mononuclear macrophages or B cells secrete HLA-DQA1 and bind to TCR to enhance antigen presentation and cytotoxic activity. Based on the above analysis, the main mechanism of anti-tumor immune regulation in RCC is adaptive immunity. Up-regulated expression of HLA-DQA1 promotes binding to TCR molecules, which is conducive to antigen presentation and the specific killing effect of T cell toxicity in tumor cells. There was no difference in the expression of the specific tumor immunoregulatory factor HLA-DQA1 in the normal epithelium of paracancerous tissues between RCC and LCC (**Figure 6**).

In LCC, we identified high expression of IL-34, which predicted a good prognosis. IL-34 is highly expressed in LCC. IL-34 is a cytokine that promotes the differentiation and viability of monocytes and macrophages through the colony stimulating factor-1 receptor (CSF1R). IL-34 is produced by different cell populations, including endothelial cells, adipocytes, neurons, macrophages, fibroblasts, and epithelial cells (30). IL-34 is secreted by M0 macrophages, endothelial cells, or other cells, and the high expression of IL-34 in LCC promotes cytokine secretion and NK cell activity. IHC analysis of biomarkers of M1 and M2, CD80 and CD163, showed that the expression of differentiated macrophages M1 and M2 was higher in RCC. The CD80 infiltration was higher in RCC but there was no statistical difference, which may be because CD80 in tissue was not a very specific M1 marker. TCGA cohort revealed that macrophage M0 expression in LCC was significantly higher than in RCC. While the scope of macrophage activation states is complex, it is generally simplified into two categories: M1 classically activated macrophages or alternatively activated M2 macrophages. M1 macrophages promote a pro-inflammatory Th1 responses, upregulate genes involved in antigen processing and presentation, and have the potential to participate in antitumor immunity. Although M2 macrophages play a critical role in normal immune function, certain subsets of M2 macrophages also play a critical role in promoting tumor progression (17, 31). It can be inferred from the above results that in LCC, the main mechanism of antitumor immune regulation is the innate immune mechanism. Flow cytometry showed that there was greater secretion of the cytokines IL-6 and IL-10. Cytokines function in many fundamental processes for life and disease including immunity, inflammation, regeneration, angiogenesis and so on. Circulating cytokines levels are multifactorial and other sources apart from the tumor contribute to this, it relates to the immune status of the patient (32). Studies have shown that IL6 and IL10 can directly or indirectly stimulate NK cell activity, thus exerting anti-tumor effects (33, 34), which is consistent with previous studies indicating that NK cells are highly expressed in LCC and significantly correlated with the prognosis of patients (11). Meanwhile, cytokines such as IL-6 and IL-10 can further stimulate the secretion of IL-34 by tumor cells and immune cells and promote the expression of IL-34. In the normal epithelium of paracancerous tissues in RCC and LCC, the expression of the innate immune regulator IL-34 was also higher in LCC than in RCC, which indicated that IL-34 was involved in the nontumor-specific innate immune mechanism, which was dominant in LCC but suppressed in RCC (**Figure 6**).

Furthermore, the role of IL-34 in tumors has been controversial in different studies. IL-34 has been reported to promote tumorigenesis through autocrine or paracrine effects (30). IL-34 has also been reported to have an antiregulatory effect in tumors. For example, IL-34 hinders the proliferation, clonogenicity, and motility of glioblastoma cells and promotes the differentiation of monoblastic leukemia cells into monocyte-like cells (35, 36). Analysis of IL-34 RNA expression and breast cancer subtypes showed that high IL-34 expression correlated with a better prognosis in luminal and HER2 subtypes and a worse prognosis in the basal one (37). It can be seen that the regulatory role of IL-34 in tumors is extremely complex, which may be related to tumor type and tumor location. Many studies have reported that IL-34 is a marker of poor prognosis in CRC, while the results of this study showed that IL-34 is a marker of poor prognosis only in LCC (38, 39).

In conclusion, this study screened and identified differentially expressed immunoregulatory factors in RCC and LCC, combined with the detection and analysis of cell infiltration in the immune microenvironment. Specific immunoregulatory factor HLA-DQA1 in RCC and immunoregulatory factor IL-34 in LCC were identified and their functions and effects involved in different immunoregulatory mechanisms were further analyzed. Undeniably, there are still some limitations in this study that need further research and discussion. In Future studies, the specific mechanisms by which HLA-DQA1 and IL-34 affect survival will be further explored, and the influence of HLA-DQA1 activation on the antitumor effect of RCC will be further investigated, as well as the possible role of IL-34 as a cytokine therapy in LCC. It is hoped that these two factors can provide a theoretical basis for the precise immunotherapy of RCC and LCC.

## Data Availability Statement

The original contributions presented in the study are included in the article/**Supplementary Material**. Further inquiries can be directed to the corresponding authors.

## Ethics Statement

The studies involving human participants were reviewed and approved by Ethic Committee of Nanjing Drum Tower Hospital. The patients/participants provided their written informed consent to participate in this study.

## Author Contributions

HY, LY, and YQ planned and designed the experiments and wrote the manuscript. DJ performed the flow cytometry, prepared the tissue microarray and performed the IHC staining. WC performed the bioinformatics analysis. GH and GM collected the specimens. All authors contributed to the article and approved the submitted version.

## Funding

The present study was supported by grants from the National Natural Science Foundation of China (NSFC) (grant nos. 81671541, 81701545 and 82071738), Clinical Medicine Science & Technology Project of Jiangsu Province of China (grant no. BL2013024), The Obstetrics and Gynecology Disease Biobank, Jiangsu Biobank of Clinical Resources (BM2015004), and The Open Project of Jiangsu Biobank of Clinical Resources (SBK202006003, SBK202006001, SBK202006002).

## Conflict of Interest

The authors declare that the research was conducted in the absence of any commercial or financial relationships that could be construed as a potential conflict of interest.

## Publisher’s Note

All claims expressed in this article are solely those of the authors and do not necessarily represent those of their affiliated organizations, or those of the publisher, the editors and the reviewers. Any product that may be evaluated in this article, or claim that may be made by its manufacturer, is not guaranteed or endorsed by the publisher.

## Supplementary Material

Supplementary Table 1 Comparison of pathological features between RCC and LCC patients in database.

Supplementary Table 2 Comparison of pathological features between RCC and LCC clinical patients.

Supplementary Table 3 Flow cytometry results of Th1/Th2/Th17 cytokines.

Supplementary Figure 1 Survival analysis of the other DEGs.

Supplementary Figure 2 Comparison of infiltration degree of different immune cells in LCC and RCC.
