# Supplementary material for: Differential Expression and Prognostic Correlation of Immune Related Factors Between Right and Left Side Colorectal Cancer
Source: Front Oncol. 2022 Jul 22;12:845765. doi: 10.3389/fonc.2022.845765 (PMC9353740; doi:10.3389/fonc.2022.845765)
Supplement: Supplementary Table 1 — Comparison of pathological features between RCC and LCC patients in database. [file Table_1.docx]

**Supplementary Table 1**

Comparison of pathological features between RCC and LCC patients in database.

| **Class** |  | **LCC(n=207)** | **RCC(n=318)** | **p** |
| --- | --- | --- | --- | --- |
| **Age** |  |  |  |  |
|  | ≥70y | 80 | 177 |  |
|  | ＜70y | 127 | 141 | 0.0001 |
| **Gender** |  |  |  |  |
|  | male | 109 | 164 |  |
|  | female | 98 | 154 | 0.8079 |
| **Pathologic stage** |  |  |  |  |
|  | Ⅰ | 31 | 52 |  |
|  | Ⅱ | 71 | 140 |  |
|  | Ⅲ | 65 | 85 |  |
|  | Ⅳ | 37 | 34 | 0.1753 |
| **Pathology T stage** |  |  |  |  |
|  | T1 | 4 | 5 |  |
|  | T2 | 38 | 52 |  |
|  | T3 | 147 | 215 |  |
|  | T4 | 18 | 46 | 0.1517 |
| **Pathology N stage** |  |  |  |  |
|  | N-0 | 110 | 202 |  |
|  | Non N-0 | 97 | 116 | 0.0179 |
| **Pathology M stage** |  |  |  |  |
|  | M-0 | 149 | 237 |  |
|  | Non M-0 | 57 | 75 | 0.3533 |
| **Mucus secretion** |  |  |  |  |
|  | yes | 13 | 53 |  |
|  | no | 193 | 265 | 0.0005 |
| **Microsatellite stability** |  |  |  |  |
|  | MSI | 47 | 142 |  |
|  | MSS | 154 | 176 | 0.0001 |
