# Supplementary material for: Differential Expression and Prognostic Correlation of Immune Related Factors Between Right and Left Side Colorectal Cancer
Source: Front Oncol. 2022 Jul 22;12:845765. doi: 10.3389/fonc.2022.845765 (PMC9353740; doi:10.3389/fonc.2022.845765)
Supplement: Supplementary Table 2 — Comparison of pathological features between RCC and LCC clinical patients. [file Table_2.docx]

**Supplementary Table 2**

Comparison of pathological features between RCC and LCC clinical patients.

| **Class** |  | **LCC(n=50)** | **RCC(n=50)** | **p** |
| --- | --- | --- | --- | --- |
| **Age** |  |  |  |  |
|  | ≥70y | 14 | 22 |  |
|  | ＜70y | 36 | 28 | 0.0478 |
| **Gender** |  |  |  |  |
|  | male | 31 | 28 |  |
|  | female | 19 | 22 | 0.5419 |
| **Pathologic stage** |  |  |  |  |
|  | Ⅰ | 5 | 5 |  |
|  | Ⅱ | 11 | 27 |  |
|  | Ⅲ | 28 | 16 |  |
|  | Ⅳ | 6 | 2 | 0.9999 |
| **Pathology T stage** |  |  |  |  |
|  | T1 | 0 | 0 |  |
|  | T2 | 7 | 6 |  |
|  | T3 | 40 | 39 |  |
|  | T4 | 3 | 5 | 0.7447 |
| **Pathology N stage** |  |  |  |  |
|  | N-0 | 16 | 32 |  |
|  | Non N-0 | 34 | 18 | 0.0007 |
| **Pathology M stage** |  |  |  |  |
|  | M-0 | 44 | 50 |  |
|  | Non M-0 | 6 | 0 | 0.0058 |
| **Mucus secretion** |  |  |  |  |
|  | yes | 6 | 8 |  |
|  | no | 44 | 42 | 0.2822 |
| **Microsatellite stability** |  |  |  |  |
|  | MSI-H | 9 | 20 |  |
|  | MSS | 41 | 30 | 0.0153 |
