# Supplementary figures and images for: Differential Expression and Prognostic Correlation of Immune Related Factors Between Right and Left Side Colorectal Cancer
Source: Front Oncol. 2022 Jul 22;12:845765. doi: 10.3389/fonc.2022.845765 (PMC9353740; doi:10.3389/fonc.2022.845765)

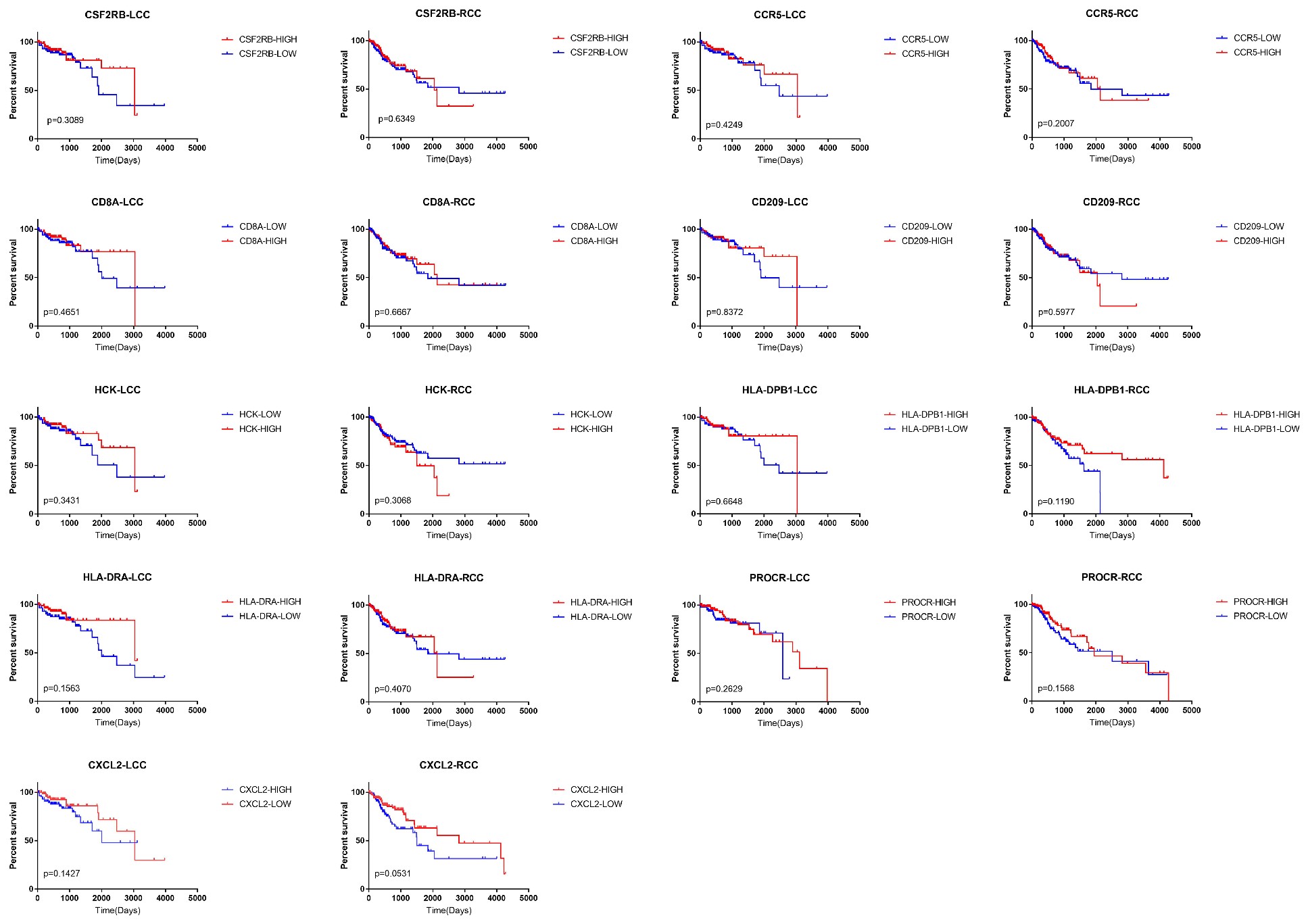

Supplement: Supplementary Figure 1 — Survival analysis of the other DEGs. [file Image_1.jpeg]

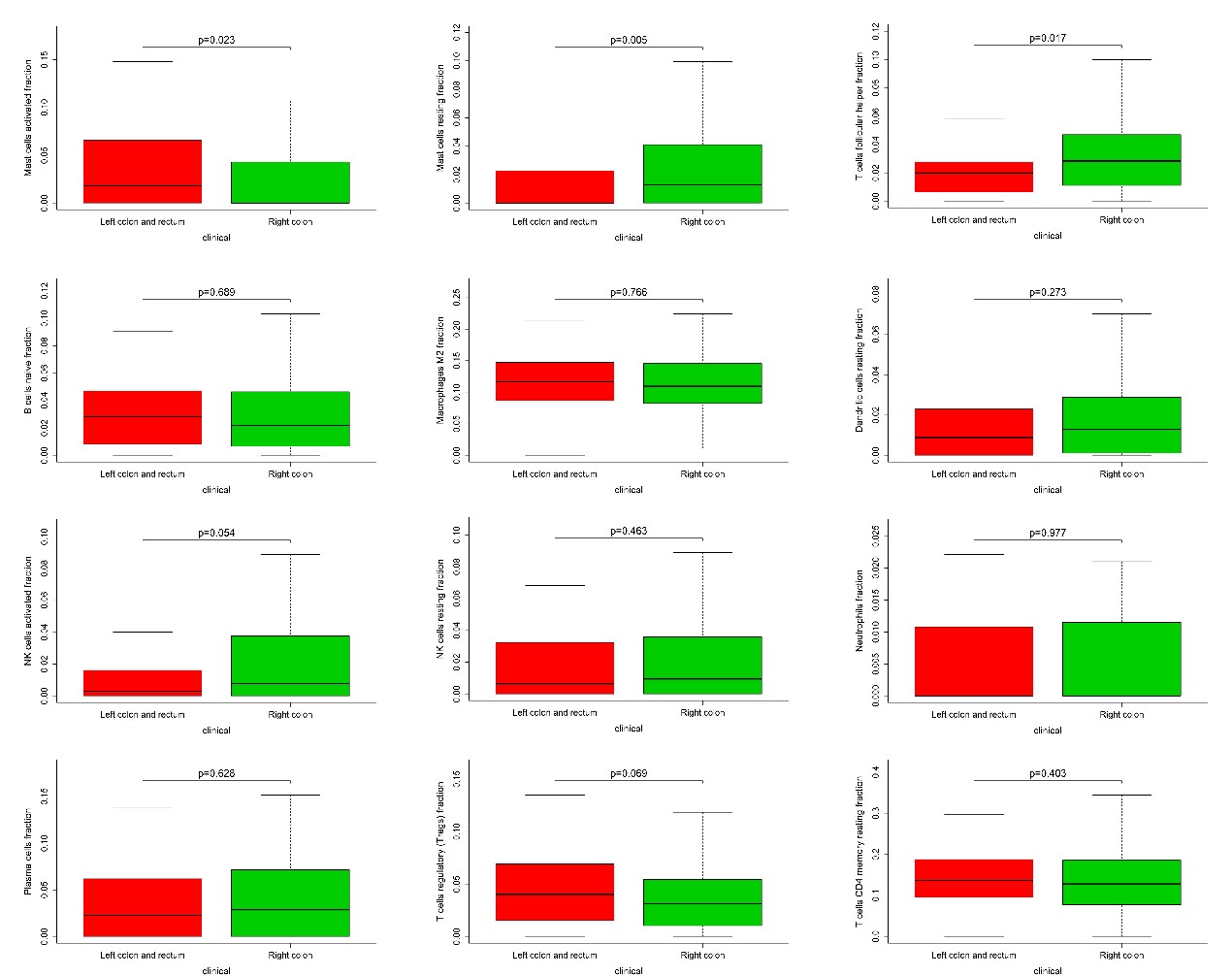

Supplement: Supplementary Figure 2 — Comparison of infiltration degree of different immune cells in LCC and RCC. [file Image_2.jpeg]
